# Case Report: Carcinoma en Cuirasse in a Middle-Aged Woman Mimicking Postirradiation Morphea

**DOI:** 10.3389/fonc.2021.747123

**Published:** 2021-10-21

**Authors:** Haiqing Wang, Chengbei Bao, Ting Gong, Chao Ji

**Affiliations:** ^1^Department of Dermatology, The First Affiliated Hospital of Fujian Medical University, Fuzhou, China; ^2^Central Laboratory, The First Affiliated Hospital of Fujian Medical University, Fuzhou, China

**Keywords:** carcinoma en cuirasse, breast cancer, cutaneous metastases, postirradiation morphea, radiation dermatitis

## Abstract

Breast carcinoma en cuirasse (CeC) is an extremely rare form of cutaneous metastases of breast cancer, characterized by diffuse sclerodermoid induration of the skin. It may be difficult to distinguish CeC from some skin diseases, including postirradiation morphea, inflammatory breast cancer, radiation dermatitis, and other cutaneous metastases, but it can be easily discerned by histology. Because of the small number of documented cases, the treatment consensus has not been clearly defined. Here, we show a 45-year-old woman with grade III infiltrating ductal carcinoma manifesting as CeC to the chest wall. Early diagnosis and treatment are essential to prevent the catastrophic natural progression of this rare malignancy.

## Introduction

Carcinoma en cuirasse (CeC), also known as scirrhous carcinoma (1), is a rare form of cutaneous metastasis of breast cancer. It appears as diffuse cutaneous and subcutaneous carcinomatous infiltration of the mammary region that may invade the chest wall and abdomen (2). In most cases, CeC tends to develop after the initial intervention of the primary tumor (3). Due to the rarity of cases, clinical data on this type of malignancy are limited. This makes differentiating CeC from other skin disorders or other cutaneous metastases hard. We describe here a case of a patient who developed CeC and discuss the literature about clinical manifestation, differential diagnosis, and treatment approach of this aggressive malignancy.

## Case Report

**Figure 1:**
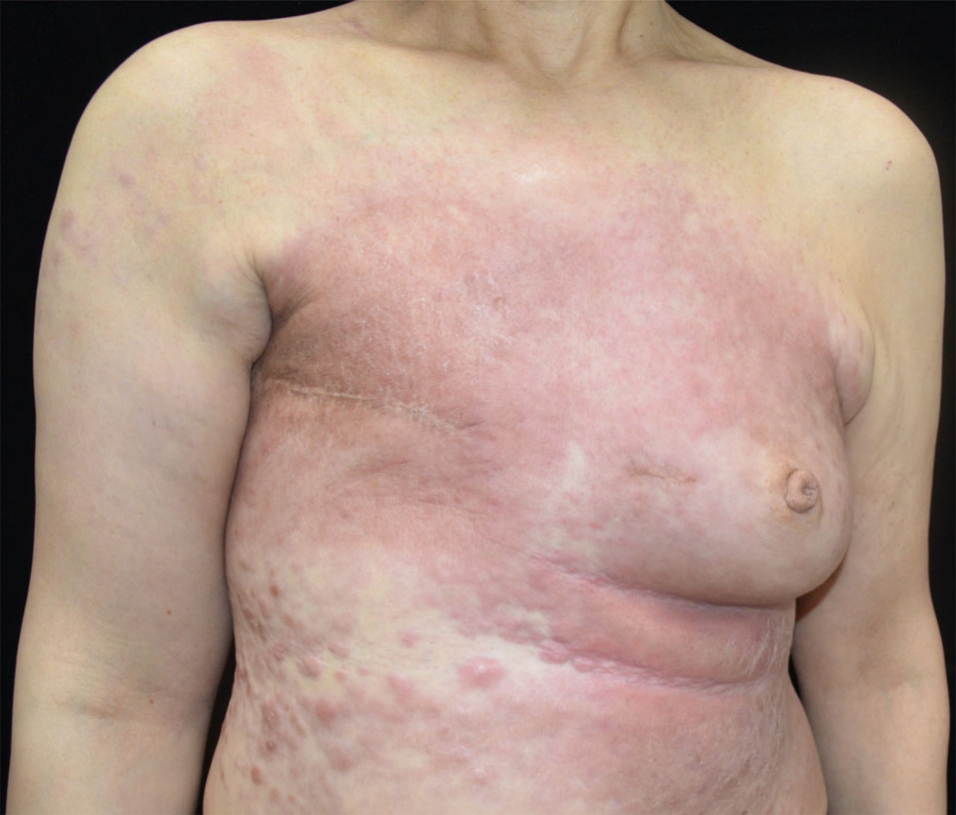
Clinical picture of the patient. Elevated, keloid-like, fine nodules coalescing into diffuse sclerodermoid plaques in a background of erythema were found on the right upper arm, chest wall, and abdomen.

A 45-year-old female presented in August 2017 with an enlarging right breast mass. She was nulliparous with a 20-pack-year smoking history. Fine-needle aspiration and excisional biopsy were consistent with grade III infiltrating ductal carcinoma. She received several cycles of radio- and chemotherapy followed by right modified radical mastectomy. In May 2021, she was referred to us with a painful, sclerodermoid lesion over the chest wall. On examination, the chest wall and abdomen were diffusely indurated with erythematous nodules coalescing into diffuse sclerodermoid plaques in a background of erythema, and the right upper arm was also involved (**Figure 1**). On the basis of her previous history of radiotherapy as well as the current clinical presentation, a diagnosis of postirradiation morphea was firstly considered.

**Figure 2:**
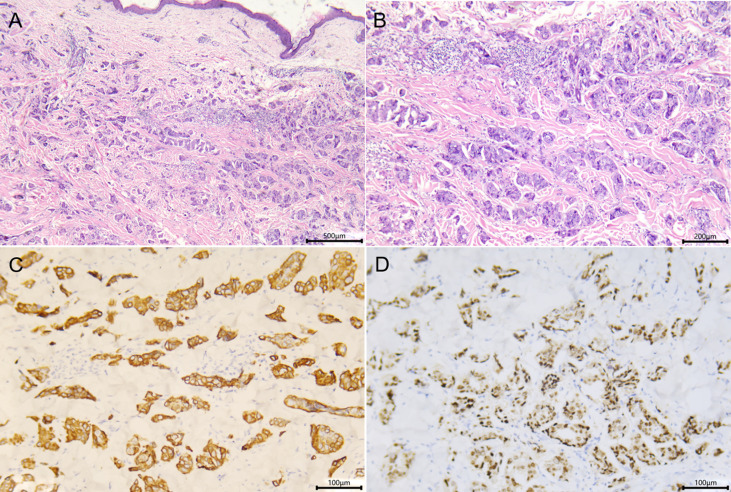
Skin histopathology of the patient. **(A, B)** Biopsy specimen of the chest showed single files or small clusters of monomorphous atypical cells with a cytoplasm rich in vacuoles seen in the dermis [**(A)** hematoxylin and eosin, ×50; **(B)** hematoxylin and eosin, ×100]. **(C, D)** Immumohistochemical staining showed the tumor cells were CK7+ **(C)** and ER+ **(D)** (×200).

A biopsy of chest lesion showed a proliferation of monomorphous atypical tumor cells with a cytoplasm rich in vacuoles, presenting a single-file pattern or in small clusters (**Figures 2A, B**). Immunohistochemical staining revealed atypical cells were positive for cytokeratin 7 and estrogen receptor, but cytokeratin 20, progesterone receptor, and GCDFP-15 were negative (**Figures 2C, D**). Taken together, the clinical and histopathological findings were consistent with CeC. Bone and computed tomography scans of the chest and abdomen showed no distant disease. She was referred to the oncology department and received palliative treatment with carboplatin and gemcitabine, at the time of writing this case.

## Discussion

Breast cancer is the most frequently diagnosed malignancy in women throughout the world (4), and cutaneous metastasis of breast cancer is associated with advanced stage of cancer and poorer prognosis (5). Median survival of cutaneous metastases in breast cancer patients was 13.8 months with a 3.1% 10-year survival rate (6).

CeC is a rare clinicopathologic type of skin metastasis with a few cases reported to date. It is more common in patients with tumor recurrence after mastectomy, radiation, or chemotherapy and characterized by diffuse sclerodermoid induration of the skin (7). The clinical presentation of CeC includes two stages. It begins to manifest as scattered, flesh-colored nodules in a background of erythematous skin surface and eventually coalesces into a sclerodermoid plaque (8).

The specific clinical manifestations of CeC lead to confusion with some skin diseases, including postirradiation morphea, inflammatory breast cancer, radiation dermatitis, and other cutaneous metastases. Postirradiation morphea is an uncommon complication after radiation therapy which often demonstrates as an erythematous indurated plaque and may be mistaken for metastatic carcinoma (9). In the beginning, postirradiation morphea was listed as diagnostic consideration; however, it can be discerned by histology. Histopathologically, it is characterized by thick collagen bundles, loss of adventitial fat, and atrophic eccrine glands, and unlike findings in CeC, there is no atypical tumor cell infiltrate. Radiation dermatitis and inflammatory breast cancer are associated with inflammatory changes distinct from CeC (10).

There is no treatment consensus for CeC due to the rarity of this type of malignancy, but it has been reported that chemotherapy, local irradiation, skin graft, hormonal antagonists, and non-steroidal anti-inflammatory drug therapy showed some success (5, 11, 12).

We hereby report a case of CeC developing as a presentation of invasive ductal breast cancer to highlight the phenomenon of cutaneous metastasis. The diagnosis of CeC relies on clinical and histopathologic features to distinguish it from other entities described herein. Although rare, early detection, diagnosis, and intervention of CeC are critical for dermatologists.

## Data Availability Statement

The original contributions presented in the study are included in the article/**Supplementary Material**. Further inquiries can be directed to the corresponding authors.

## Ethics Statement

The studies involving human participants were reviewed and approved by the Medical Technology Clinical Application Ethics Committee of the First Affiliated Hospital of Fujian (No. [2021]105). Written informed consent was obtained from the individual(s) for the publication of any potentially identifiable images or data included in this article.

## Author Contributions

HW and CB were responsible for collecting the clinical data and drafting the article. CJ and TG took charge of revising the article. All authors contributed to the article and approved the submitted version.

## Conflict of Interest

The authors declare that the research was conducted in the absence of any commercial or financial relationships that could be construed as a potential conflict of interest.

## Publisher’s Note

All claims expressed in this article are solely those of the authors and do not necessarily represent those of their affiliated organizations, or those of the publisher, the editors and the reviewers. Any product that may be evaluated in this article, or claim that may be made by its manufacturer, is not guaranteed or endorsed by the publisher.
