# Supplementary figures and images for: Case Report: Carcinoma en Cuirasse in a Middle-Aged Woman Mimicking Postirradiation Morphea
Source: Front Oncol. 2021 Oct 21;11:747123. doi: 10.3389/fonc.2021.747123 (PMC8566709; doi:10.3389/fonc.2021.747123)

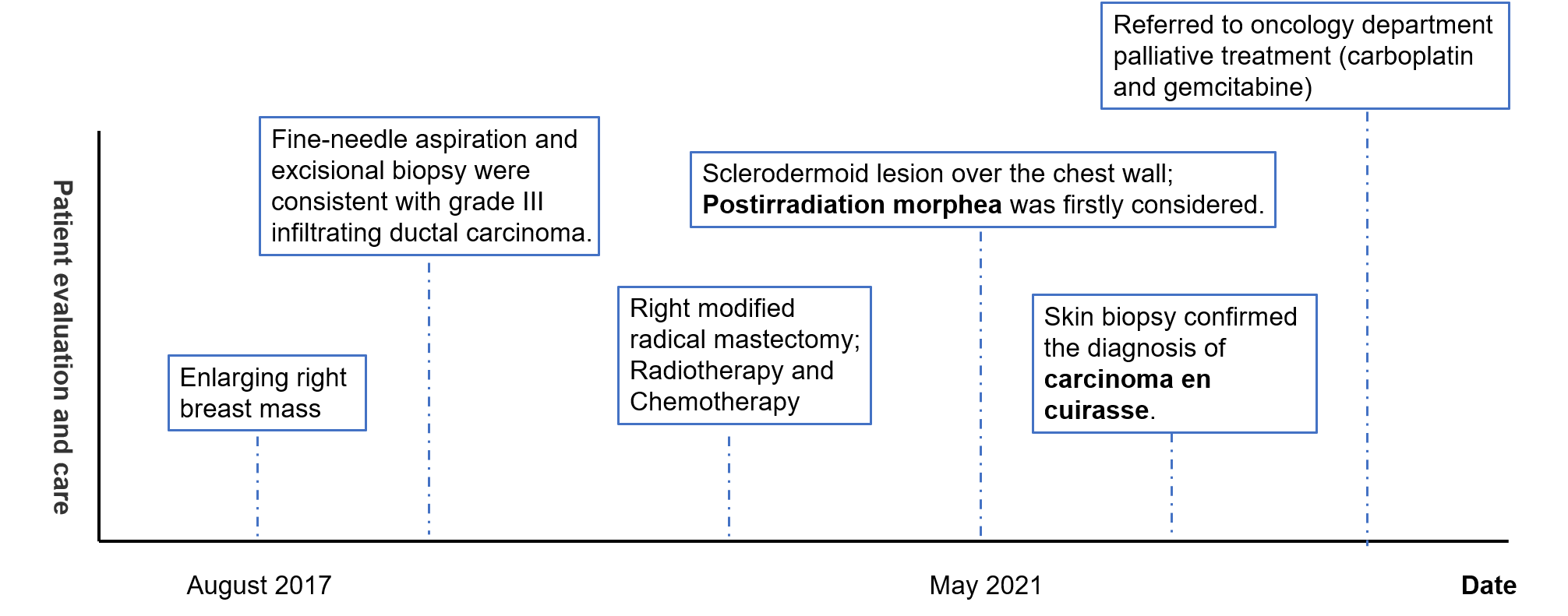

Supplement: Supplementary file 1 [file Image_1.tif]
